# Manipulation of New Fluorescent Magnetic Nanoparticles with an Electromagnetic Needle, Allowed Determining the Viscosity of the Cytoplasm of M-HeLa Cells

**DOI:** 10.3390/ph16020200

**Published:** 2023-01-29

**Authors:** Iliza Ramazanova, Maxim Suslov, Guzel Sibgatullina, Konstantin Petrov, Svetlana Fedorenko, Asiya Mustafina, Dmitry Samigullin

**Affiliations:** 1 Kazan Institute of Biochemistry and Biophysics, FRC Kazan Scientific Center, Russian Academy of Sciences, Lobachevsky Str., 2/31, 420111 Kazan, Russia; 2 Arbuzov Institute of Organic and Physical Chemistry, FRC Kazan Scientific Center, Russian Academy of Sciences, Arbuzov Str., 8, 420088 Kazan, Russia; 3 Department of Radiophotonics and Microwave Technologies, Kazan National Research Technical University Named after A.N. Tupolev-KAI, 10 K. Marx St., 420111 Kazan, Russia

**Keywords:** magnetic nanoparticles, electromagnetic needle, HeLa cells, viscosity

## Abstract

Magnetic nanoparticles (MNPs) have recently begun to be actively used in biomedicine applications, for example, for targeted drug delivery, in tissue engineering, and in magnetic resonance imaging. The study of the magnetic field effect on MNPs internalized into living cells is of particular importance since it allows a non-invasive influence on cellular activity. There is data stating the possibility to manipulate and control individual MNPs utilizing the local magnetic field gradient created by electromagnetic needles (EN). The present work aimed to demonstrate the methodological and technical approach for manipulating the local magnetic field gradient, generated by EN, novel luminescent MNPs internalized in HeLa cancer cells. The controlling of the magnetic field intensity and estimation of the attractive force of EN was demonstrated. Both designs of EN and their main characteristics are also described. Depending on the distance and applied voltage, the attractive force ENs ranged from 0.056 ± 0.002 to 37.85 ± 3.40 pN. As a practical application of the presented, the evaluation of viscous properties of the HeLa cell’s cytoplasm, based on the measurement of the movement rate of MNPs inside cells under impact of a known magnetic force, was carried out; the viscosity was 1.45 ± 0.04 Pa·s.

## 1. Introduction

Non-invasive action on the cells and tissues of the body is currently one of the promising areas of research in biology and medicine [1,2,3]. Finding ways to exert a controlled influence on cell activity is an important problem in nanotechnology and nanomedicine [4,5]. Particularly promising are the studies related to the development and study of ferromagnetic nanoparticles, the properties of which can be controlled at a distance using magnetic fields [6,7,8]. Magnetic nanoparticles (MNPs) have recently begun to be actively used in biomedicine applications for targeted drug delivery, tissue engineering, and magnetic resonance imaging [9,10]. The magnetic field is non-invasive and can penetrate deeply into living tissues. However, to overcome the weak interaction between magnetic fields and biological tissues, magnetic fields must be converted into mechanical forces, heating, or changes in electrical potential [11,12,13,14]. Superparamagnetic iron oxide nanoparticles can be considered a promising tool for such translation mediated by magnetic fields. Due to its deep penetration into living tissues, the magnetic field can noninvasively affect the properties of cells with the internalized MNPs [15]. This effect can be manifested in the form of a thermal effect due to heating induced by a high-intensity alternating magnetic field or the so-called magnetothermia [16]. In addition, the effect on cells can be mediated by mechanical action on cell organelles and membranes during the movement of MNPs when a magnetic field is applied [11,17]. This mechanical action can lead to the activation of physiological processes that change cellular activity. In particular, this is the activation of ion channels and membrane receptors [18,19,20] and the destruction of cell membranes when strong magnetic fields are applied [9,21]. The mechanical movement of the MNPs internalized into the cell cytoplasm under the influence of a magnetic field is used to assess the viscosity properties of the cell’s cytoplasm [22]. The viscoelasticity of the cytoplasm is one of the important parameters of cellular processes, such as the transport of vesicles and other intracellular compartments. It is also important for cellular locomotion. The viscoelastic properties of the cytoplasm change at the stage of cell division and can characterize the stages of division [23]. Electromagnetic tweezers are used to measure the viscoelastic properties of the cytoplasm and generate an electromagnetic field to influence the MNPs [22]. An electromagnetic needle (EN) can be considered one of the modifications of electromagnetic tweezers used for local manipulation of MNPs, which allows targeted action on individual cells with the internalized MNPs [24,25]. Using this approach, it was possible to control the cell growth of retinal ganglion cells that were cultured on gold and nickel meshes and in neurons using the membrane-bound MNPs [26,27]. This experimental approach made it possible to obtain the directed growth of neurons in the primary culture of the chicken brain [28].

The studies listed above did not control or describe the movement of nanoparticles in a magnetic field inside living cells. In the present study, we used the already reported MNPs with a fluorescent label. These MNPs are non-toxic and have good cell penetrating ability [29]. Due to the presence of a fluorescent label, it became possible to use confocal microscopy methods, which made it possible to characterize the amount of movement of nanoparticles internalized into HeLa cancer cells in a magnetic field gradient created by an electromagnetic needle. In this study, we also estimated the magnitude of the attraction force of the electromagnetic needle at different magnetic field inductions. Using the proposed approach to measure the movement of MNPs in the cytoplasm, we measured the viscosity of the cell’s cytoplasm using fluorescent magnetic particles and the method of confocal microscopy. The application of confocal microscopy methods that allow one to monitor the effects of the local magnetic field gradient of the EN on the MNPs internalized in cells can serve as a powerful tool for studying signaling processes in living cells.

## 2. Results

### 2.1. Force Calibration of the Electromagnetic Needle

**Figure 1:**
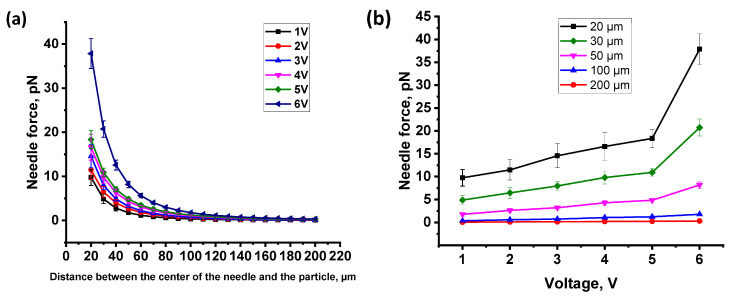
Force calibration of the electromagnetic needle. (**a**) Distance dependence of the force on the particle for 6 different coil voltages (1–6 V); (**b**) Force-versus-voltage curves for the five distances indicated in the inset showing a relationship between the coil voltage and the force on the magnetic nanoparticles (MNPs) (at a distance of 20, 30, 50, 100, and 200 μm between the tip of the electromagnetic needle (EN) and the MNPs).

At the first stage of the work, the attractive force of the manufactured EN was calculated according to the method given in the Materials and Methods section. For the detailed description of experimental procedure and calculations see the Exp. Section 4.4. As a result, the average value of the attractive force of the EN was found for the voltage range of 1–6 V with a step of 1 V, and graphs of the dependencies of the needle attraction force on the distance between the center of the needle and the particle at various voltages were plotted (Figure 1a), along with the dependence of attracting force of the EN depending on the applied voltage (Figure 1b). It was found that with a decrease in the distance between the EN and the particle, as well as with an increase in the applied voltage, the attractive force of the EN increases. The results are listed in Table 1.

### 2.2. Effect of the Magnetic Field Gradient on MNPs Loaded into HeLa Cancer Cells

**Figure 2:**
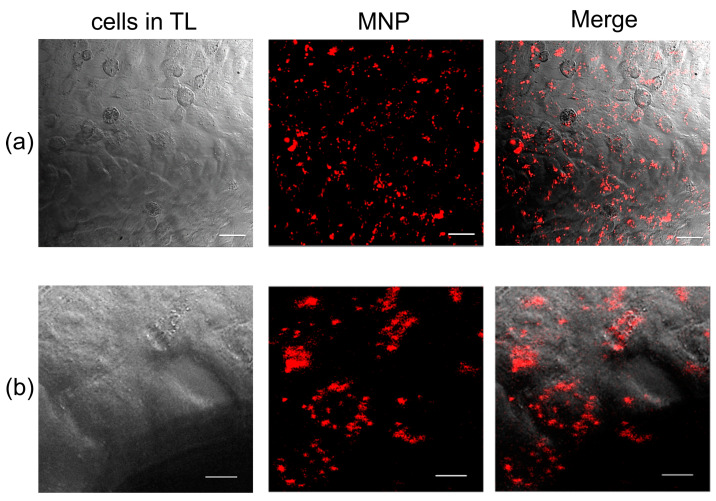
HeLa cancer cells loaded with MNPs in transmitted light (TL). Line (**a**) scale bar–30 µm, line (**b**) scale bar–10 µm.

In the next series of experiments, using EN, we studied the effect of the magnetic field gradient on MNPs loaded into HeLa cancer cells. Cultured cells were loaded with nanoparticles according to the method described by S. Fedorenko [29]. As a result, 100 ± 1% of the cells in culture were internalized with MNPs (Figure 2), which is consistent with the results previously obtained with these nanoparticles on motor neurons [29].

**Figure 3:**
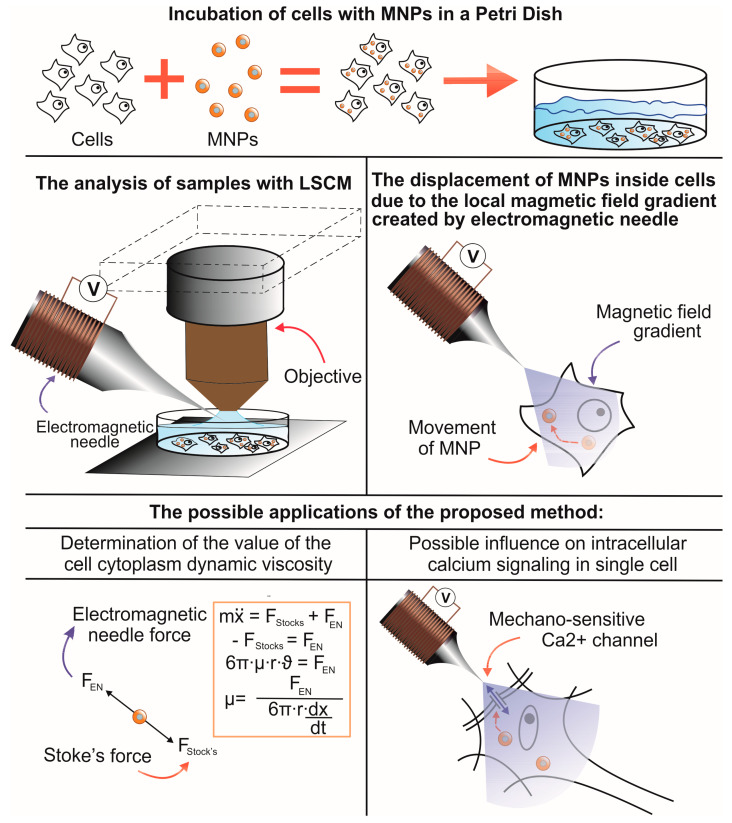
Scheme diagram of the presented method and its possible application.

**Figure 4:**
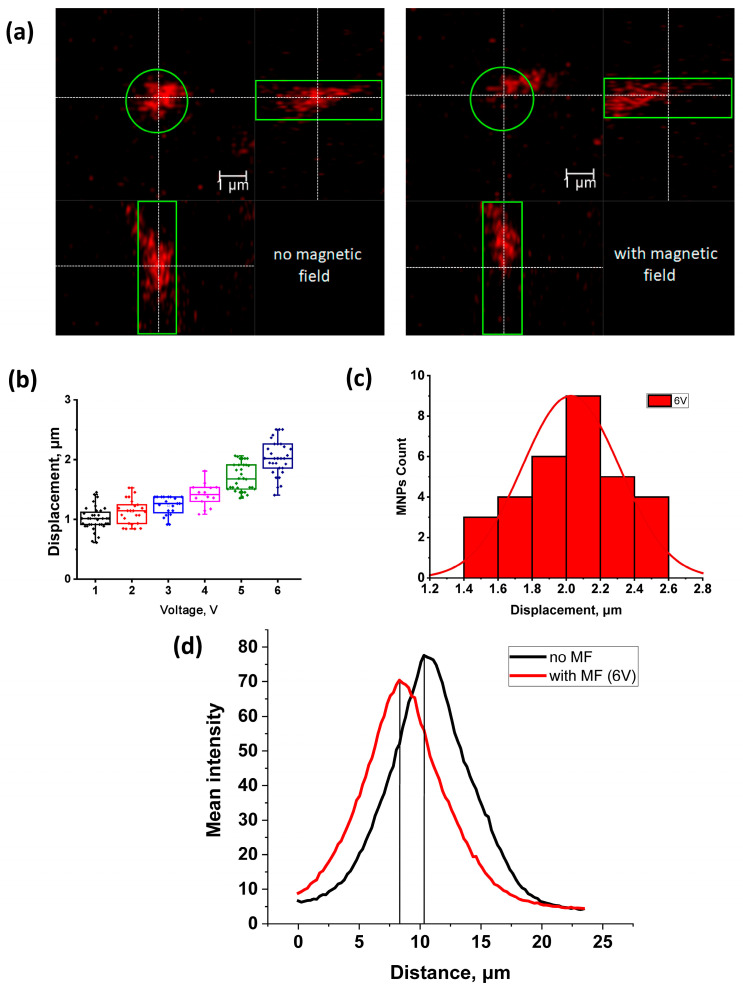
Movement of the MNPs inside cells after application of magnetic field gradient by EN. (**a**) Orthogonal projections (xy, xz and yz) of MNPs in a HeLa cancer cell: no magnetic field–without the influence of the magnetic field of the EN, with magnetic field–under the influence of the magnetic field of the EN (U = 6 V), stimulation with a magnetic field was carried out for 3 min. (**b**) Mean ± SEM and SD of the displacements of MNPs at different voltages 1–6 V. (**c**) Distribution of distances between peaks of the fluorescence intensity from MNPs, before and after the application of the magnetic field (U = 6 V). (**d**) MNP fluorescence intensity peaks in the z-axis in (**a**) before (black) and after (red) magnetic field application (U = 6 V).

Cells internalized with nanoparticles were placed on the object stage of a confocal microscope, and the ENs were brought to the cell along with nanoparticles using a micromanipulator under visual control (Figure 3). The average distance from the EN to the cell was 200 μm. The distance was determined using the confocal microscope software. The lens was focused on the tip of the EN, and, using a Z-drive, the cell with MNPs was focused. The Z-drive scale could be used to determine the distance from the needle to the cage. Then the magnetic field was turned on for 180 s, and video images were recorded in the XYZt mode. The recording was carried out for a voltage range of 1–6 V, for which the force of attraction of the EN was previously calculated (Table 1). In cells, nanoparticles are presented as fluorescent light spots with different diameters (Figure 2). This is due to the fact that MNPs loaded into cells form conglomerates of different shapes and sizes ranging from tens of nanometers to several micrometers. As mentioned in Section 4.5, to calculate the cytoplasmic viscosity of HeLa cancer cells we chose conglomerates of nanoparticles of predominantly spherical shape and about 1 µm in radius. The movement of the MNPs in the cells was detected after the voltage was applied (Figure 4).

After recording the video, the experimental data were processed, and the displacement of the MNPs was analyzed depending on the magnitude of the applied force. The analysis was performed by shifting the peaks of MNP fluorescence intensity in cancer cells using the Leica TCS SP5 confocal laser microscope software in the absence and presence of a magnetic field generated by the EN at various voltages of 1–6 V with a step of 1 V (Figure 4). A relationship was also noted between the applied voltage and the displacement of MNP in HeLa cancer cells: as the voltage increased from 1 to 6 V, the displacement increased from 1.01 ± 0.03 to 2.03 ± 0.05 μm, respectively. The measurement results are listed in Table 1. The magnetic field induction at the EN tip was assessed using a constructed device based on a Hall sensor [30], and the residual magnetization was removed by applying a damped alternating magnetic field (Table 1).

According to the method described in Section 4.5, the average dynamic viscosity of the cytoplasm of HeLa cancer cells was calculated at a voltage of 6 V. As a result of calculations, the average dynamic viscosity of the cytoplasm of HeLa cancer cells was 1.45 ± 0.04 Pa·s (n = 10).

## 3. Discussion

In this paper, we present a highly deterministic manipulation technique with MNPs inside cells. We characterized and calculated the distance of movement of MNPs inside cells under the action of a local magnetic field gradient caused by EN. Then, the trajectory and speed of movement of the particles were recorded and these parameters were used to calculate the viscosity. It is well known that the movement of MNPs was recorded using light microscopes and conventional video cameras [22,24,25]. In the present study, we used a similar approach, but unlike previous works, we used new fluorescent MNPs that can be used in fluorescence confocal microscopy [29]. The use of the confocal microscopy technique using fluorescent MNPs makes it possible to conduct studies in three dimensions and with a higher resolution than in conventional optical microscopy. The presence of a fluorescent label in MNPs allows the use of nanoparticles of smaller diameter, which have a better penetrating ability into cells. Nanoparticles penetrate cells mainly through the endocytosis pathway [31]. Different pathways of internalization, intracellular transport, and intracellular localization of nanoparticles can vary depending on their coverage and size. Nanoparticles can be localized inside cells in endosomes and lysosomes, and can penetrate into mitochondria [31,32]. The surface exposed amino groups of the MNPs prompt their escape from late endosomes and lysosomes [33], thus, facilitating the exit of the MNPs into the cell cytoplasm. Depending on the coverage, MNPs can enter the cell cytosol and remain in the cytoplasm in a free state [34,35]. It has been shown that the MNPs used in our study can be in the cell in a free state and affect the operation of ion channels through mechanical influence [29]. In our study, we showed that these MNPs internalized 100 ± 1% of the HeLa cells, which is compatible with previous data obtained on motoneurons [29]. Due to the presence of the label ([Ru(dipy)3]2+), they can be easily detected in cells. As a result of the applied technique, we obtained the value of the average viscosity of the cytoplasm of HeLa cancer cells, which was 1.45 ± 0.04 Pa·s (n = 10). In early studies performed using the method of spectrally resolved fluorescence measurements of a porphyrin-dimer-based molecular rotor, the obtained values of the viscosity of the cytoplasm of HeLa cells were in the range of 0.05–0.2 Pa·s [36,37]. The work of J.-F.Berret et al. represents the local viscoelasticity of living cells measured by rotational magnetic spectroscopy. Using this method, it was found that the viscosity of the cytoplasm of HeLa cancer cells is in the range from 10–100 Pa·s [38]. The values we obtained fit well within these ranges. This may also indicate that MNPs may be in the cytoplasm in a free state. It should be also noted that the cell viscosity was measured with different methods, which can also affect the results obtained. The literature data describe a fairly wide range of viscosities of the cell cytoplasm, which can differ by orders of magnitude [22,23]. It is known that the viscosity of the cell cytoplasm depends on many parameters, including the density of cell compartments, cytoskeleton, etc. [39]. It has been shown that the viscosity properties of the cytoplasm also change depending on the stage of cell development [23]. The obtained dynamic viscosity values are in good agreement with the data obtained by other methods. Thus, the proposed method can be used to measure the viscosity of the cytoplasm of different cells under different physiological conditions. Compared with other methods, the method we used has a number of advantages. It does not require special equipment for loading cells with nanoparticles, does not depend on temperature as much as the method based on Brownian motion, and does not require complex specialized equipment as does the method of rotational magnetic spectroscopy. The attractive force of MNPs created by an electromagnetic needle at various voltages from 1 to 6 V was measured. A relationship was noted between the applied voltage and the displacement of MNPs in cells: with an increase in voltage from 1 to 6 V, the displacement increased from 1.01 ± 0.03 to 2.03 ± 0.05 μm, respectively. The magnitude of the attractive force that we obtained (at a voltage of 1–6 V) amounted from 0.056 ± 0.002–0.302 ± 0.027 pN (at a distance of 200 μm between the EN and the particle) to 9.75 ± 1.82–37.85 ± 3.4 pN (at a distance of 20 μm between the EN and the particle) and fits into the range of forces that were described and used by other authors in their works [9,26]. They showed that particles, when exposed to magnetic forces of similar orders, are able to cause activation of ion channels and receptors on the membrane. At the same time, due to the influence of the magnetic field on MNPs located on the filopodia membrane of the axon growth cone of retinal ganglion cells, it is possible to control the direction of filopodia growth [26]. Thus, using MNPs and the magnetic field gradient generated by EN, various effects on cells can be performed to study cell signaling.

## 4. Materials and Methods

### 4.1. Magnetic Nanoparticles

In the present study, novel superparamagnetic SPION MNPs with an iron oxide core and a fluorescent complex [Ru(dipy)3]2+ are used. The MNPs were synthesized in accordance using the previously published procedure [29]. These are amino-modified silica nanoparticles (78 nm in diameter) doped with iron oxide cores. The surface-exposed amino groups facilitate the cell internalization of the MNPs. The encapsulation of [Ru(dipy)3]2+ phosphorescent complexes into a silica matrix, which allows control of the penetration of the MNPs into cells and monitoring of the behavior of the MNPs inside cells using a confocal fluorescent microscope due to the bright emission in the red wavelength range upon the excitation (450 nm). The magnetic properties of the MNPs are ensured by the inclusion of superparamagnetic iron oxide cores 17.1 nm in size in the silica matrix [29]. The detailed characterization of MNPs is available in Supplementary Materials (Figure S1, Table S1).

Ru, Si, and Fe were identified in the colloids using the simultaneous inductively coupled plasma optical emission spectrometry (ICP-OES) model iCAP 6300 DUO by Varian Thermo Scientific Company equipped with a CID detector. This spectrometer enables the simultaneous measurement of peak heights within the 166 to 867 nm range. The optical resolution is less than 0.007 nm to 200 nm. The working frequency is 27.12 MHz. Together, the radial and axial view configurations enable optimal peak height measurements with suppressed spectral noises.

### 4.2. Cells

HeLa cells were obtained from the collection of cell cultures of the Institute of Cytology, Russian Academy of Sciences. The cells were seeded in the amount of 1 × 10^6^ cell/well on poly-L-lysine coated cover glasses 24 × 24 mm placed in 6-well plates in DMEM containing 1% L-glutamine, 0.1% gentamicin, and 10% FBS. Cells were cultured in a 5% CO_2_ atmospheric condition at 37 °C. Cells were loaded with the MNPs on the third day after seeding. The MNPs were added directly to the culture medium. The final concentration of the MNPs was 20 µg/mL. 24 h after the introduction of nanoparticles, the cells were washed and placed in Dulbecco’s phosphate-buffered saline solution. The dynamic behavior of MNPs into cells was analyzed in living cells using a Leica TCS SP5 confocal laser microscope.

### 4.3. Electromagnetic Needle

**Figure 5:**
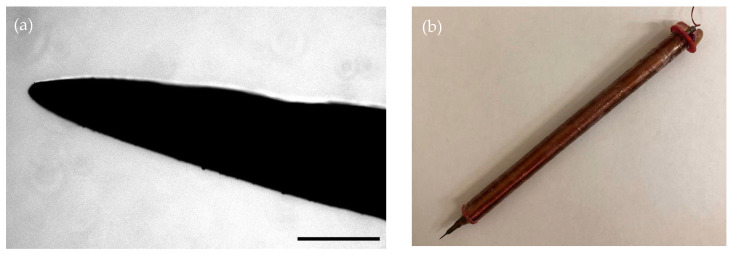
Electromagnetic needle. (**a**) View of the tip of the needle after etching, scale bar–150 µm; (**b**) Electromagnetic needle in PLA body with copper winding wire.

The EN was fabricated in a manner similar to the design described in [24,25]. A stainless-steel rod with a diameter of 4 mm was used as the EN. Using electrolysis in 10% HCl solution, the tip of the rod was reduced (etched) to 40 μm in diameter (Figure 5a). To generate a magnetic field, the needle was placed in a PLA (polylactide) housing printed on an Anet A6l 3D printer (using the Fusion360 program for modeling), with a copper winding wire with a diameter of 0.2 mm (3300 turns) wound around it (Figure 5b). The voltage on the EN coil was generated using a laboratory power supply B5-44A. To move the EN, it was fixed on the PPM5000 PiezoPatch micromanipulator (WPI, Sarasota, FL, USA), which made it possible to move the EN with high resolution (1 nm/step).

### 4.4. Calculation of the EN’s Force

Calculations to find the attractive force of the EN were carried out experimentally using the mathematical model described in [24,25]. To do this, the tip of the needle was placed in distilled water with the MNPs added to it at a ratio of 20 μg/mL. Subsequently, the MNPs were attracted to each other in the water, forming conglomerates of various shapes and sizes; all subsequent calculations were performed with microscopic MNP conglomerates with predominantly spherical shape and about 1 µm in radius. Firstly, this is due to the fact that in this study the Stokes method was used to calculate the dynamic viscosity of the cell cytoplasm, which is based on the model of spherical particle motion in a liquid. Secondly, it is related to the convenience of observing the movement of particles in the cell cytoplasm. The needle was connected to a B5-44A direct current source, and the voltage was applied in the range of 1–6 V with a step of–1 V. Residual magnetization in EN was removed by applying a damped alternating magnetic field. A video of the attraction of MNPs at various voltages from 1–6 V was recorded, which was further divided into frames. Frame rate fps = 3.74625 1/s, and time between frames t = 1/f = 0.2669336 s. On each frame, the position of the particles was fixed, the distance between the tip of the needle and the particles was found, and the particle sizes were also determined. The movement of the particles was determined by the difference in distance between the particle and the tip of the needle between adjacent frames. The velocities ϑ for each particle were calculated using formula (1):

(1) $$\vartheta =\frac{\Delta x}{\Delta t}$$

where Δx is the movement of the particle, and Δt is the time of movement of the particle. The motion of a particle can be modeled according to the law (2):

(2) $$m\ddot{x}=F_{Stokes}+F_{EN}$$

where m is the mass of the particle, $\ddot{x}$ is the acceleration of the particle, $F_{Stocks}$ is the Stokes force, and $F_{EN\;}$is the attractive force of the EN. We can consider the acceleration $\ddot{x}$= 0 on an infinitely small section, and, therefore, we obtain Equation (3):

(3) $$-F_{Stokes}=F_{EN}\;$$

We calculate the force of the needle for each particle according to the formula (4):

(4) $$F_{EN}=6\pi \cdot \mu \cdot r\cdot \vartheta$$

where μ is the viscosity of the liquid in which the particles are located, and *r* is the radius of the particle.

Plots of force versus distance were plotted for particles at voltages from 1–6 V (μ for water was taken as $10^{-3}$ Pa∙s or $10^{-3}$ kg/m∙s). The resulting graphs were approximated similarly to Formula (5) described in [24,25]:

(5) $$\;F_{m}=\frac{2\pi }{3}d_{p}^{3}\rho_{p}M_{p}\frac{\beta M_{n}^{2}}{{\left(4\beta \delta +1\right)}^{3}}$$

where $\;F_{m}$ is the magnetic force depending on the distance between the particle and the needle tip; dp, $\rho_{p}$ and $M_{p}$ are the diameter, density, and mass magnetization of the particle, respectively; *β* is a coefficient related to the pole shape; Mn is the needle core magnetization; and *δ* is the distance between the needle tip and the particle.

This Equation (5) was rewritten in the form (6), according to which further approximation was carried out:

(6) $$F=\frac{\sigma d^{3}\beta }{{\left(4\beta \delta +1\right)}^{3}},\;$$

where *σ* is a parameter proportional to the density, mass magnetization of the particle and the needle core magnetization. In our calculations, this parameter was selected for each conglomerate separately in order to approximate the obtained graphs of the dependence of the attracting forces of the *EN* on the distance.

### 4.5. Calculation of the Cytoplasmic Viscosity of HeLa Cancer Cells

The viscosity of the cytoplasm was calculated using the Stokes Formula (7):

(7) $$\;\mu =\frac{F_{EN}}{6\pi \cdot r\cdot \frac{dx}{dt}},$$

where *μ* is the viscosity of the medium in which the MNPs are located, $F_{EN}$ is the *EN* force at a distance of 200 μm between the needle tip and the particle, *r* is the particle radius, *dx* is the displacement of the MNPs inside the cells, and *t* is the magnetic field stimulation time. According to the microscopic measurements, the radius of the nanoparticle fluorescence spot was taken as 1 µm, and the magnetic stimulation time was 180 s (3 min).

### 4.6. Microscopic Technique

To calculate the attractive force of the EN, a Nikon Inverted Microscope Eclipse TE300 fluorescent microscope equipped with a fluorescence filter cube, mercury lamp, and an AxioCamMRm ZEISS camera was used. Fluorescence was detected using excitation filter wavelengths with 450–490 nanometers and a 500-nanometers LP emission filter. AxioVisionRel 4.8. software was used for visualization, and the screen video was recorded using the Bandicam 2.3.1.840 program. Observations were performed using a 40× objective (0.60 NA; Nikon).

Measurements related to the study of the movement of MNPs inside HeLa cancer cells were carried out using a Leica TCS SP5 confocal laser microscope using an argon laser with a wavelength of 488 nm. Observations were performed using a 20× water immersion objective (1.00 NA; Leica Microsystems, Heidelberg, Germany). Nanoparticles were visualized by detecting fluorescence in the range from 600–640 nm. The XYZt confocal microscope mode was used. Video images were recorded with 512 × 512 resolution using a photomultiplier in the LAS AF software (Leica Microsystems, Heidelberg, Germany). The height of Z-Stacks of optical slices was 0.25 µm. Leica TCS SP5 orthogonal section instruments were used to determine the position of the maximum fluorescence intensity. Image analysis was performed in the LAS X software (Leica Microsystems, Heidelberg, Germany). To determine changes in MNP localization, we analyzed the position of the maximum fluorescence intensity from a single nanoparticle and its movement along the z axis before and after the application of a magnetic field.

Transmission electron microscopy images of silica nanoparticles were obtained using a Hitachi HT7700 apparatus (Japan). The images were acquired under an accelerating voltage of 100 kV. Samples were ultrasonicated in absolute ethanol for 10 min and then dispersed on 200-mesh copper grids with continuous formvar support films.

## 5. Conclusions

Thus, the described method makes it possible to manipulate nanoparticles locally inside individual cells and allows for dosed mechanical action on cell membranes and organelles. Using this approach, it was possible to determine the viscosity of the cell cytoplasm. This method will allow mechanical action on cell membranes and potentially trigger cell signaling. Mechanical action on cell membranes in excitable cells can promote the activation of mechano-sensitive channels and, as a result, promote the entry of calcium [40], which, in turn, can lead to the launch of various intracellular signal chains [41,42]. In the works presented earlier, such an impact was performed using a constant magnetic field, which cannot be quickly dosed and selects individual cells for exposure [29,43]. Using EN, it is possible to dose and locally create a magnetic field gradient and act on individual cells or on sections of the membrane of individual cells. For example, it is possible to trigger signaling processes locally in individual nerve cells [44] or even in individual dendrites [7,11]. Using the effect of the local gradient of the EN magnetic field on MNPs internalized into cells can serve as a powerful tool for studying signaling processes in living cells.

## Figures and Tables

**Table 1:** Relationship between the applied voltage on the coil and the magnetic field induction, displacement of MNPs in HeLa cancer cells, and the average value of the magnetic force.

Voltage, V	Current, mA	Needle Magnetic Field Induction, mT	Displacement Value, µm	The Value of the Average Force (at a Distance of 200 μm between the EN and the Particle), pN	The Value of the Average Force (at a Distance of 20 μm between the EN and the Particle), pN
1	29	3.3 ± 0.3	1.01 ± 0.03	0.056 ± 0.002	9.75 ± 1.82
2	59	6.0 ± 0.6	1.13 ± 0.04	0.103 ± 0.017	11.47 ± 2.02
3	86	7.4 ± 0.6	1.23 ± 0.03	0.127 ± 0.019	14.55 ± 2.61
4	116	8.1 ± 0.3	1.41 ± 0.05	0.198 ± 0.033	16.59 ± 2.96
5	144	9.5 ± 0.3	1.70 ± 0.04	0.236 ± 0.050	18.30 ± 2.02
6	169	10.7 ± 0.3	2.03 ± 0.05	0.302 ± 0.027	37.85 ± 3.40

## Data Availability

Data is contained within the article.

## Supplementary Materials

The following supporting information can be downloaded at: https://www.mdpi.com/article/10.3390/ph16020200/s1, Figure S1: TEM image of MNPs; Table S1: The Si:Fe:Ru molar ratios of MNPs calculated from ICP-OES (inductively coupled plasma optical emission spectrometry) data, diameter of MNPs (d_TEM_) evaluated from TEM, average size (d), electrokinetic potential (ζ), polydispersity index (PDI) from DLS measurements in water.
