# Supplementary material for: Manipulation of New Fluorescent Magnetic Nanoparticles with an Electromagnetic Needle, Allowed Determining the Viscosity of the Cytoplasm of M-HeLa Cells
Source: Pharmaceuticals (Basel). 2023 Jan 29;16(2):200. doi: 10.3390/ph16020200 (PMC9965334; doi:10.3390/ph16020200)
Supplement: Supplementary file 1 [file pharmaceuticals-16-00200-s001.zip › pharmaceuticals-2164068-supplementary.pdf]

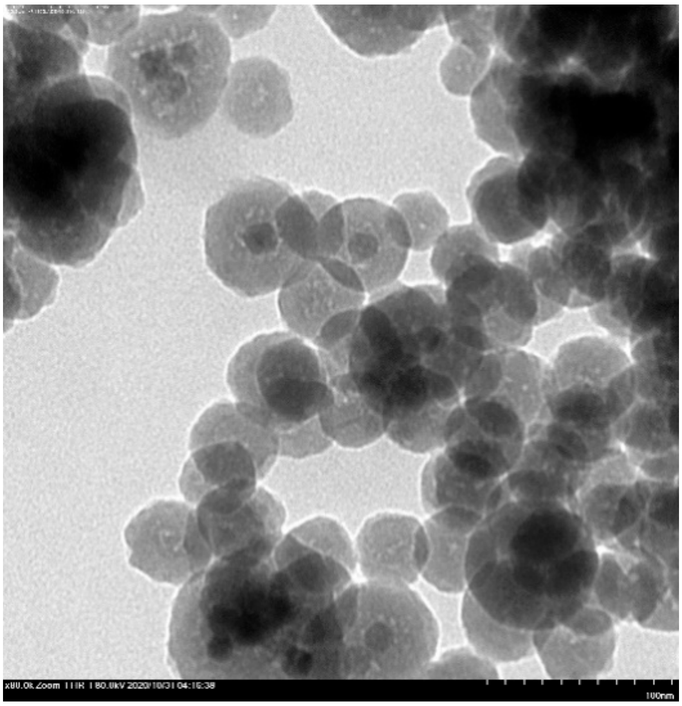

Figure S1. TEM image of MNPs.

Table S1. The Si:Fe:Ru molar ratios of MNPs calculated from ICP-OES (inductively coupled plasma optical emission spectrometry) data, diameter of MNPs (d_{TEM}) evaluated from TEM, average size (d), electrokinetic potential (ζ)**,** polydispersity index (PDI) from DLS measurements in water.

Sample	Molar ratio of Si:Fe:Ru (ICP-OES)	$C_{NH2\text{-}groups}$ (mM) per 1 g L^{-1} dispersion	d_{TEM}/ nm	DLS data		
				d/nm	PDI	ζ/ mV
	1:0.07:0.011	0.275	78±14	900±100	0.882	+14
